# Dichlorido(2,9-dimeth­oxy-1,10-phenanthroline-κ^2^ *N*,*N*′)zinc(II)

**DOI:** 10.1107/S160053680902491X

**Published:** 2009-07-04

**Authors:** Cao-Yuan Niu, Hui Su, Lei Meng, Chun-Hong Kou

**Affiliations:** a College of Sciences, Henan Agricultural University, Zhengzhou 450002, People’s Republic of China

## Abstract

In the crystal structure of the title compound, [ZnCl_2_(C_14_H_12_N_2_O_2_)], the Zn^II^ center is four-coordinated by two N atoms from one 2,9-dimeth­oxy-1,10-phenanthroline ligand and two Cl atoms. The coordination geometry is distorted tetra­hedral, as the Zn—N bond distances are shorter than the Zn—Cl distances, and the Cl—Zn—N and Cl—Zn—Cl bond angles are much larger than the N—Zn—N angle. For the ligand, the O and C atoms of the meth­oxy groups are almost in the plane defined by the phenanthroline ring. The two O atoms deviate from the phenanthroline mean plane by 0.076 (2) and 0.084 (2) Å, and the two methyl C atoms deviate from the phenanthroline mean plane by 0.035 (3) and 0.361 (3) Å. There are medium π–π stacking interactions between two parallel phenanthroline rings with a centroid–centroid distance of 3.7860 (2) Å and a dihedral angle between the plane defined by the two parallel phenanthroline rings of 1.13 (5)°.

## Related literature

For background information, see: Majumder *et al.* (2006 ▶); Bie *et al.* (2006 ▶). For the synthesis, see: Pijper *et al.* (1984 ▶).
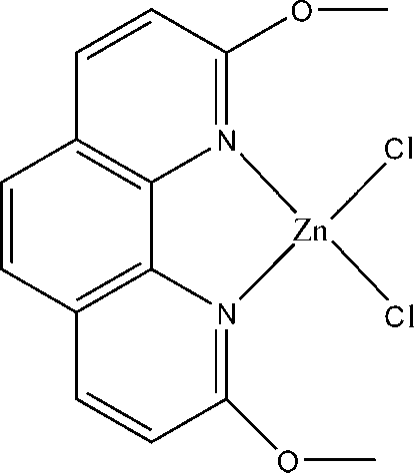


## Experimental

### 

#### Crystal data

[ZnCl_2_(C_14_H_12_N_2_O_2_)]
*M* *_r_* = 376.53
Monoclinic, 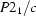

*a* = 9.0494 (8) Å
*b* = 10.3783 (9) Å
*c* = 16.3517 (14) Å
β = 99.022 (1)°
*V* = 1516.7 (2) Å^3^
*Z* = 4
Mo *K*α radiation
μ = 1.98 mm^−1^
*T* = 291 K
0.27 × 0.14 × 0.10 mm

#### Data collection

Bruker APEXII CCD detector diffractometer
Absorption correction: multi-scan (*SADABS*; Sheldrick, 1996 ▶) *T*_min_ = 0.616, *T*_max_ = 0.835
9326 measured reflections
3465 independent reflections
2917 reflections with *I* > 2σ(*I*)
*R*_int_ = 0.017

#### Refinement

*R*[*F* ^2^ > 2σ(*F* ^2^)] = 0.025
*wR*(*F* ^2^) = 0.065
*S* = 1.03
3465 reflections
192 parameters
H-atom parameters constrained
Δρ_max_ = 0.30 e Å^−3^
Δρ_min_ = −0.25 e Å^−3^

### 

Data collection: *SMART* (Siemens, 1996 ▶); cell refinement: *SAINT* (Siemens, 1994 ▶); data reduction: *SAINT*; program(s) used to solve structure: *SHELXS97* (Sheldrick, 2008 ▶); program(s) used to refine structure: *SHELXL97* (Sheldrick, 2008 ▶); molecular graphics: *SHELXL97* and *DIAMOND* (Brandenburg, 2005 ▶); software used to prepare material for publication: *SHELXL97*.

## Supplementary Material

Crystal structure: contains datablocks I, globsl. DOI: 10.1107/S160053680902491X/fj2228sup1.cif

Structure factors: contains datablocks I. DOI: 10.1107/S160053680902491X/fj2228Isup2.hkl

Additional supplementary materials:  crystallographic information; 3D view; checkCIF report

## Figures and Tables

**Table:** 

Zn1—N1	2.0659 (14)
Zn1—N2	2.0911 (14)
Zn1—Cl1	2.2007 (6)
Zn1—Cl2	2.2219 (6)

**Table:** 

N1—Zn1—N2	80.58 (6)
N1—Zn1—Cl1	113.27 (4)
N2—Zn1—Cl1	120.54 (4)
N1—Zn1—Cl2	110.66 (4)
N2—Zn1—Cl2	108.06 (4)
Cl1—Zn1—Cl2	117.82 (2)

**Fig. 1.**
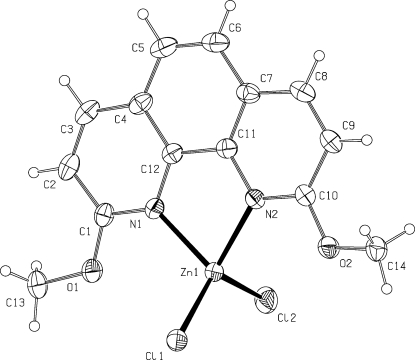
A view of the ZnII coordination environment in (I), showing the atom-labelling scheme. Displacement ellipsoids are drawn at the 30% probability level and H atoms are shown as small spheres of arbitrary radii.

**Fig. 2.**
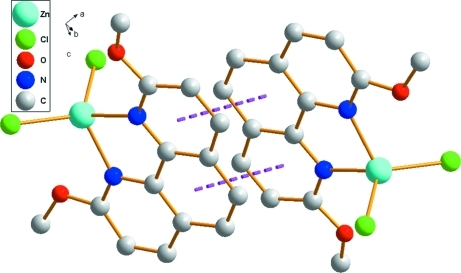
Packing diagram showing the π-π interaction (purple dotted line). All H atoms have been omitted for clarity.

**Fig. 3.**
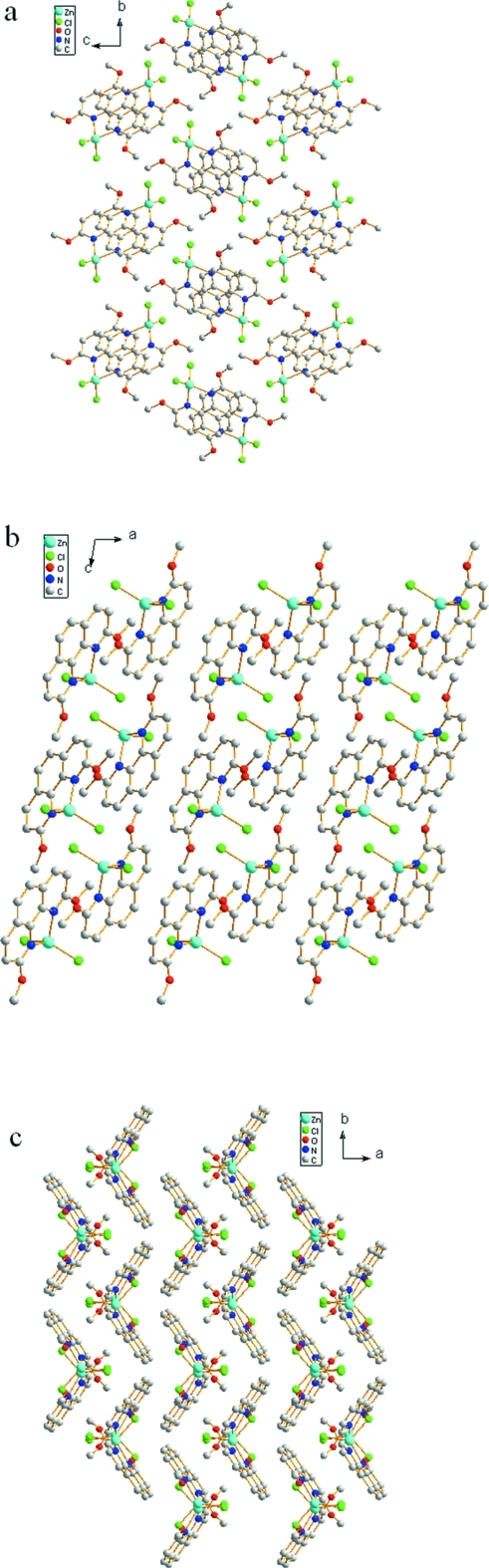
Diagrams showing the three-dimensional packing forms along three crystallographic directions.

## supplementary crystallographic information

### Comment

The compound 1,10-phenanthroline was reported to be used to synthesize some potential strong luminescent materials with d^10^ metals. It can be predicted that the title compound that composed of a derivative of 1,10-phenanthroline and a d^10 ^metal zinc would possess strong ligand to ligand or metal perturbed ligand to ligand emissions (Majumder *et al.*, 2006; Bie, *et al.*, 2006). 2,9-Dimethoxy-1,10-phenanthroline and 2,9-Diethoxy-1,10-phenanthroline as derivatives of 1,10-phenanthroline were synthesized at early time and they possess antimycoplasmal activity in the presence of copper (Pijper, *et al.*, 1984).

The title compound (I) is a mononuclear zinc(II) complex of 2,9-dimethoxy-1,10-phenanthroline (shown as Fig.1). The zinc metal centre is four coordinated to two nitrogen atoms (N1, N2) from the 1,10-phenanthroline ring and two independent chlorine atoms (Cl1, Cl2), defining a deformed tetrahedron coordination geometry around the metal center. The Zn—Cl bond distances are 2.2007 (6) and 2.2219 (6) Å, which are longer than the Zn—N bond distances from 2.0659 (14) to 2.0911 (14) Å. The Cl—Zn—N and Cl—Zn—Cl bond angles are at the range of 108.06 (4) to 120.54 (4) °, which are larger than that of N—Zn—N [80.58 (6)°]. Furthermore, there are medium π-π stackings between two parallel phenanthroline rings from two symmetry-related monomers with the centroid-to-centroid distances of about 3.7860 (2) Å and dihedral angle of 1.13 (5) ° (Fig. 2). For the ligand, two methoxy groups are basically coplanar to the phenanthroline ring. Two oxygen atoms deviate from the phenanthroline plane by 0.076 (2) and 0.084 (2) Å, and two methyl carbon atoms deviate from the phenanthroline plane by 0.035 (3) and 0.361 (3) Å.

Three-dimensional supramolecular structure of the title compound is formed *via* the above-mentioned π-π stackings and weak van der waals interactions. Some interesting packings along three crystallographic directions can be seen from Fig. 3.

### Experimental

The organic ligand 2,9-dimethoxy-1,10-phenanthroline was prepared according to the procedure of literature (Pijper, *et al.*, 1984). The slow evaporation of mixture of the ligand (0.022 g, 0.1 mmol) and zinc dichloride (0.014 g, 0.1 mmol) in 30 ml me thanol afforded suitable colourless block crystals in about 7 days (yield 45%).

### Refinement

Carbon-bound H atoms were positioned geometrically and refined using a riding model [C—H = 0.93 Å and *U*_iso_(H) = 1.2 *U*_eq_(C) for aromatic H atoms; C—H = 0.96 Å and *U*_iso_(H) = 1.5 *U*_eq_(C) for methyl H atoms;]. The final difference Fourier map had a highest peak at 0.85 Å from atom Cl2 and a deepest hole at 0.59 Å from atom Cl2, but were otherwise featureless.

### Crystal data

**Table:**

[ZnCl_2_(C_14_H_12_N_2_O_2_)]	*F*(000) = 760
*M**_r_* = 376.53	*D*_x_ = 1.649 Mg m^−^^3^
Monoclinic, *P*2_1_/*c*	Mo *K*α radiation, λ = 0.71073 Å
Hall symbol: -P 2ybc	Cell parameters from 4023 reflections
*a* = 9.0494 (8) Å	θ = 2.3–27.9°
*b* = 10.3783 (9) Å	µ = 1.98 mm^−^^1^
*c* = 16.3517 (14) Å	*T* = 291 K
β = 99.022 (1)°	Block, colorless
*V* = 1516.7 (2) Å^3^	0.27 × 0.14 × 0.10 mm
*Z* = 4	

### Data collection

**Table:**

Bruker APEXII CCD detector diffractometer	3465 independent reflections
Radiation source: fine-focus sealed tube	2917 reflections with *I* > 2σ(*I*)
graphite	*R*_int_ = 0.017
φ and ω scans	θ_max_ = 27.5°, θ_min_ = 2.3°
Absorption correction: multi-scan (*SADABS*; Sheldrick, 1996)	*h* = −7→11
*T*_min_ = 0.616, *T*_max_ = 0.835	*k* = −13→13
9326 measured reflections	*l* = −21→21

### Refinement

**Table:**

Refinement on *F*^2^	Primary atom site location: structure-invariant direct methods
Least-squares matrix: full	Secondary atom site location: difference Fourier map
*R*[*F*^2^ > 2σ(*F*^2^)] = 0.025	Hydrogen site location: inferred from neighbouring sites
*wR*(*F*^2^) = 0.065	H-atom parameters constrained
*S* = 1.03	*w* = 1/[σ^2^(*F*_o_^2^) + (0.0298*P*)^2^ + 0.4767*P*] where *P* = (*F*_o_^2^ + 2*F*_c_^2^)/3
3465 reflections	(Δ/σ)_max_ = 0.001
192 parameters	Δρ_max_ = 0.30 e Å^−^^3^
0 restraints	Δρ_min_ = −0.25 e Å^−^^3^

### Special details

**Table:**

Geometry. All e.s.d.'s (except the e.s.d. in the dihedral angle between two l.s. planes) are estimated using the full covariance matrix. The cell e.s.d.'s are taken into account individually in the estimation of e.s.d.'s in distances, angles and torsion angles; correlations between e.s.d.'s in cell parameters are only used when they are defined by crystal symmetry. An approximate (isotropic) treatment of cell e.s.d.'s is used for estimating e.s.d.'s involving l.s. planes.
Refinement. Refinement of *F*^2^ against ALL reflections. The weighted *R*-factor *wR* and goodness of fit *S* are based on *F*^2^, conventional *R*-factors *R* are based on *F*, with *F* set to zero for negative *F*^2^. The threshold expression of *F*^2^ > σ(*F*^2^) is used only for calculating *R*-factors(gt) *etc*. and is not relevant to the choice of reflections for refinement. *R*-factors based on *F*^2^ are statistically about twice as large as those based on *F*, and *R*- factors based on ALL data will be even larger.

### Fractional atomic coordinates and isotropic or equivalent isotropic displacement parameters (Å^2^)

**Table:**

	*x*	*y*	*z*	*U*_iso_*/*U*_eq_	
Zn1	0.85210 (2)	0.73107 (2)	0.142693 (13)	0.03691 (8)	
Cl1	1.07679 (6)	0.76186 (5)	0.21365 (3)	0.05122 (14)	
Cl2	0.68839 (6)	0.89157 (5)	0.13748 (4)	0.05898 (15)	
O1	0.99184 (17)	0.81556 (14)	−0.01403 (8)	0.0502 (3)	
O2	0.71476 (16)	0.58258 (13)	0.28668 (8)	0.0481 (3)	
N1	0.85558 (16)	0.65629 (14)	0.02592 (9)	0.0366 (3)	
N2	0.73776 (16)	0.55838 (14)	0.15361 (9)	0.0360 (3)	
C1	0.9193 (2)	0.70500 (19)	−0.03500 (11)	0.0405 (4)	
C2	0.9063 (3)	0.6460 (2)	−0.11341 (12)	0.0502 (5)	
H2	0.9523	0.6820	−0.1550	0.060*	
C3	0.8256 (3)	0.5361 (2)	−0.12718 (12)	0.0519 (5)	
H3	0.8160	0.4967	−0.1788	0.062*	
C4	0.7557 (2)	0.48020 (19)	−0.06404 (12)	0.0447 (4)	
C5	0.6660 (2)	0.3663 (2)	−0.07373 (14)	0.0548 (5)	
H5	0.6523	0.3232	−0.1242	0.066*	
C6	0.6011 (2)	0.3201 (2)	−0.01103 (14)	0.0540 (5)	
H6	0.5415	0.2468	−0.0192	0.065*	
C7	0.6228 (2)	0.38262 (18)	0.06829 (13)	0.0434 (4)	
C8	0.5595 (2)	0.3401 (2)	0.13704 (14)	0.0500 (5)	
H8	0.4985	0.2675	0.1319	0.060*	
C9	0.5859 (2)	0.40325 (19)	0.21063 (13)	0.0464 (5)	
H9	0.5437	0.3748	0.2557	0.056*	
C10	0.6789 (2)	0.51305 (18)	0.21712 (12)	0.0391 (4)	
C11	0.71040 (19)	0.49372 (17)	0.07990 (11)	0.0369 (4)	
C12	0.7758 (2)	0.54491 (17)	0.01209 (11)	0.0367 (4)	
C13	1.0711 (3)	0.8779 (2)	−0.07353 (13)	0.0554 (5)	
H13A	1.1445	0.8199	−0.0891	0.083*	
H13B	1.1199	0.9540	−0.0492	0.083*	
H13C	1.0014	0.9013	−0.1217	0.083*	
C14	0.6826 (3)	0.5280 (2)	0.36304 (12)	0.0536 (5)	
H14A	0.5763	0.5196	0.3604	0.080*	
H14B	0.7216	0.5834	0.4083	0.080*	
H14C	0.7285	0.4447	0.3712	0.080*	

### Atomic displacement parameters (Å^2^)

**Table:**

	*U*^11^	*U*^22^	*U*^33^	*U*^12^	*U*^13^	*U*^23^
Zn1	0.03791 (13)	0.03966 (13)	0.03454 (12)	−0.00516 (9)	0.01002 (9)	−0.00396 (8)
Cl1	0.0399 (3)	0.0714 (4)	0.0431 (3)	−0.0099 (2)	0.0087 (2)	−0.0143 (2)
Cl2	0.0501 (3)	0.0513 (3)	0.0795 (4)	0.0074 (2)	0.0226 (3)	−0.0024 (3)
O1	0.0623 (9)	0.0531 (8)	0.0391 (7)	−0.0104 (7)	0.0201 (6)	0.0019 (6)
O2	0.0561 (9)	0.0513 (8)	0.0389 (7)	−0.0155 (7)	0.0138 (6)	0.0014 (6)
N1	0.0378 (8)	0.0402 (9)	0.0323 (7)	0.0027 (6)	0.0071 (6)	−0.0009 (6)
N2	0.0342 (8)	0.0367 (8)	0.0370 (8)	−0.0032 (6)	0.0057 (6)	0.0017 (6)
C1	0.0419 (10)	0.0461 (11)	0.0342 (9)	0.0081 (8)	0.0083 (8)	0.0043 (8)
C2	0.0608 (13)	0.0582 (13)	0.0339 (10)	0.0133 (10)	0.0148 (9)	0.0022 (9)
C3	0.0647 (14)	0.0557 (13)	0.0348 (10)	0.0144 (11)	0.0057 (9)	−0.0088 (9)
C4	0.0468 (11)	0.0458 (11)	0.0392 (10)	0.0126 (9)	−0.0006 (8)	−0.0068 (8)
C5	0.0601 (13)	0.0487 (12)	0.0520 (12)	0.0071 (10)	−0.0030 (10)	−0.0183 (10)
C6	0.0510 (12)	0.0410 (11)	0.0661 (14)	−0.0020 (9)	−0.0028 (10)	−0.0123 (10)
C7	0.0364 (10)	0.0381 (10)	0.0530 (11)	0.0008 (8)	−0.0007 (8)	−0.0035 (8)
C8	0.0405 (11)	0.0411 (11)	0.0660 (14)	−0.0081 (9)	0.0011 (10)	0.0013 (10)
C9	0.0405 (11)	0.0448 (11)	0.0544 (12)	−0.0067 (8)	0.0087 (9)	0.0086 (9)
C10	0.0353 (9)	0.0400 (10)	0.0422 (10)	−0.0016 (7)	0.0065 (8)	0.0039 (8)
C11	0.0322 (9)	0.0356 (9)	0.0411 (9)	0.0038 (7)	0.0006 (7)	−0.0018 (7)
C12	0.0345 (9)	0.0377 (10)	0.0365 (9)	0.0072 (7)	0.0009 (7)	−0.0021 (7)
C13	0.0574 (13)	0.0644 (14)	0.0490 (12)	−0.0040 (11)	0.0226 (10)	0.0113 (10)
C14	0.0590 (13)	0.0624 (14)	0.0427 (11)	−0.0119 (11)	0.0179 (10)	0.0042 (9)

### Geometric parameters (Å, °)

**Table:**

Zn1—N1	2.0659 (14)	C4—C5	1.429 (3)
Zn1—N2	2.0911 (14)	C5—C6	1.347 (3)
Zn1—Cl1	2.2007 (6)	C5—H5	0.9300
Zn1—Cl2	2.2219 (6)	C6—C7	1.436 (3)
O1—C1	1.340 (2)	C6—H6	0.9300
O1—C13	1.449 (2)	C7—C11	1.395 (3)
O2—C10	1.343 (2)	C7—C8	1.410 (3)
O2—C14	1.442 (2)	C8—C9	1.358 (3)
N1—C1	1.327 (2)	C8—H8	0.9300
N1—C12	1.363 (2)	C9—C10	1.411 (3)
N2—C10	1.325 (2)	C9—H9	0.9300
N2—C11	1.368 (2)	C11—C12	1.438 (3)
C1—C2	1.409 (3)	C13—H13A	0.9600
C2—C3	1.354 (3)	C13—H13B	0.9600
C2—H2	0.9300	C13—H13C	0.9600
C3—C4	1.417 (3)	C14—H14A	0.9600
C3—H3	0.9300	C14—H14B	0.9600
C4—C12	1.401 (3)	C14—H14C	0.9600
			
N1—Zn1—N2	80.58 (6)	C7—C6—H6	119.6
N1—Zn1—Cl1	113.27 (4)	C11—C7—C8	116.32 (18)
N2—Zn1—Cl1	120.54 (4)	C11—C7—C6	119.39 (19)
N1—Zn1—Cl2	110.66 (4)	C8—C7—C6	124.29 (19)
N2—Zn1—Cl2	108.06 (4)	C9—C8—C7	121.19 (18)
Cl1—Zn1—Cl2	117.82 (2)	C9—C8—H8	119.4
C1—O1—C13	119.03 (15)	C7—C8—H8	119.4
C10—O2—C14	117.89 (15)	C8—C9—C10	118.47 (19)
C1—N1—C12	118.68 (16)	C8—C9—H9	120.8
C1—N1—Zn1	128.47 (13)	C10—C9—H9	120.8
C12—N1—Zn1	112.80 (11)	N2—C10—O2	113.56 (16)
C10—N2—C11	118.48 (16)	N2—C10—C9	122.41 (18)
C10—N2—Zn1	129.50 (13)	O2—C10—C9	124.03 (17)
C11—N2—Zn1	111.65 (11)	N2—C11—C7	123.09 (17)
N1—C1—O1	112.80 (16)	N2—C11—C12	117.31 (16)
N1—C1—C2	122.24 (19)	C7—C11—C12	119.60 (17)
O1—C1—C2	124.95 (17)	N1—C12—C4	123.06 (17)
C3—C2—C1	118.91 (19)	N1—C12—C11	117.18 (15)
C3—C2—H2	120.5	C4—C12—C11	119.76 (17)
C1—C2—H2	120.5	O1—C13—H13A	109.5
C2—C3—C4	120.96 (18)	O1—C13—H13B	109.5
C2—C3—H3	119.5	H13A—C13—H13B	109.5
C4—C3—H3	119.5	O1—C13—H13C	109.5
C12—C4—C3	116.14 (19)	H13A—C13—H13C	109.5
C12—C4—C5	119.19 (19)	H13B—C13—H13C	109.5
C3—C4—C5	124.66 (18)	O2—C14—H14A	109.5
C6—C5—C4	121.18 (19)	O2—C14—H14B	109.5
C6—C5—H5	119.4	H14A—C14—H14B	109.5
C4—C5—H5	119.4	O2—C14—H14C	109.5
C5—C6—C7	120.8 (2)	H14A—C14—H14C	109.5
C5—C6—H6	119.6	H14B—C14—H14C	109.5
			
N2—Zn1—N1—C1	−177.42 (16)	C7—C8—C9—C10	−0.1 (3)
Cl1—Zn1—N1—C1	−58.12 (16)	C11—N2—C10—O2	178.99 (16)
Cl2—Zn1—N1—C1	76.72 (16)	Zn1—N2—C10—O2	−8.7 (2)
N2—Zn1—N1—C12	5.17 (12)	C11—N2—C10—C9	−1.9 (3)
Cl1—Zn1—N1—C12	124.47 (11)	Zn1—N2—C10—C9	170.42 (14)
Cl2—Zn1—N1—C12	−100.69 (12)	C14—O2—C10—N2	−167.68 (17)
N1—Zn1—N2—C10	−179.01 (17)	C14—O2—C10—C9	13.3 (3)
Cl1—Zn1—N2—C10	69.45 (17)	C8—C9—C10—N2	1.8 (3)
Cl2—Zn1—N2—C10	−70.22 (16)	C8—C9—C10—O2	−179.18 (18)
N1—Zn1—N2—C11	−6.24 (12)	C10—N2—C11—C7	0.3 (3)
Cl1—Zn1—N2—C11	−117.78 (11)	Zn1—N2—C11—C7	−173.33 (14)
Cl2—Zn1—N2—C11	102.56 (11)	C10—N2—C11—C12	−179.91 (16)
C12—N1—C1—O1	178.81 (15)	Zn1—N2—C11—C12	6.43 (19)
Zn1—N1—C1—O1	1.5 (2)	C8—C7—C11—N2	1.3 (3)
C12—N1—C1—C2	0.5 (3)	C6—C7—C11—N2	−179.37 (17)
Zn1—N1—C1—C2	−176.81 (14)	C8—C7—C11—C12	−178.48 (17)
C13—O1—C1—N1	178.04 (17)	C6—C7—C11—C12	0.9 (3)
C13—O1—C1—C2	−3.7 (3)	C1—N1—C12—C4	−1.1 (3)
N1—C1—C2—C3	0.2 (3)	Zn1—N1—C12—C4	176.62 (14)
O1—C1—C2—C3	−177.93 (19)	C1—N1—C12—C11	178.97 (16)
C1—C2—C3—C4	−0.3 (3)	Zn1—N1—C12—C11	−3.35 (19)
C2—C3—C4—C12	−0.2 (3)	C3—C4—C12—N1	0.9 (3)
C2—C3—C4—C5	178.6 (2)	C5—C4—C12—N1	−177.93 (17)
C12—C4—C5—C6	−0.2 (3)	C3—C4—C12—C11	−179.10 (17)
C3—C4—C5—C6	−178.9 (2)	C5—C4—C12—C11	2.0 (3)
C4—C5—C6—C7	−1.4 (3)	N2—C11—C12—N1	−2.2 (2)
C5—C6—C7—C11	1.0 (3)	C7—C11—C12—N1	177.58 (16)
C5—C6—C7—C8	−179.7 (2)	N2—C11—C12—C4	177.84 (16)
C11—C7—C8—C9	−1.4 (3)	C7—C11—C12—C4	−2.4 (3)
C6—C7—C8—C9	179.3 (2)		
